# Antidiabetic and Antioxidant Impacts of Desert Date (*Balanites aegyptiaca*) and Parsley (*Petroselinum sativum*) Aqueous Extracts: Lessons from Experimental Rats

**DOI:** 10.1155/2016/8408326

**Published:** 2016-02-25

**Authors:** Nasser S. Abou Khalil, Alaa S. Abou-Elhamd, Salwa I. A. Wasfy, Ibtisam M. H. El Mileegy, Mohamed Y. Hamed, Hussein M. Ageely

**Affiliations:** ^1^Department of Physiology, Faculty of Medicine, Assiut University, Assiut 71526, Egypt; ^2^Department of Anatomy and Histology, Faculty of Veterinary Medicine, Assiut University, Assiut 71526, Egypt; ^3^Department of Internal Medicine, Faculty of Medicine, Jazan University, Jazan 82621, Saudi Arabia

## Abstract

Medicinal plants are effective in controlling plasma glucose level with minimal side effects and are commonly used in developing countries as an alternative therapy for the treatment of type 1 diabetes mellitus. The aim of this study is to evaluate the potential antidiabetic and antioxidant impacts of* Balanites aegyptiaca* and* Petroselinum sativum* extracts on streptozotocin-induced diabetic and normal rats. The influences of these extracts on body weight, plasma glucose, insulin, total antioxidant capacity (TAC), malondialdehyde (MDA) levels, and liver-pyruvate kinase (L-PK) levels were assessed. Furthermore, the weight and histomorphological changes of the pancreas were studied in the different experimental groups. The herbal preparations significantly reduced the mean plasma glucose and MDA levels and significantly increased the mean plasma insulin, L-PK, and TAC levels in the treated diabetic groups compared to the diabetic control group. An obvious increase in the weight of the pancreas and the size of the islets of Langerhans and improvement in the histoarchitecture were evident in the treated groups compared to untreated ones. In conclusion, the present study provides a scientific evidence for the traditional use of these extracts as antidiabetic and antioxidant agents in type 1 diabetes mellitus.

## 1. Introduction

Type 1 diabetes mellitus (T1DM) occurs in childhood and is characterized by T-cell autoimmune disease mediated destruction of insulin secreting *β* cells in the pancreatic islets. This destructive process results in severe insulin deficiency that in turn leads to hyperglycemia [1–3].

T1DM remains a major global health problem, especially in developing countries, in spite of the availability of many antidiabetic drugs because they have limited efficacy and certain adverse effects. This leads to increased demand of research on antidiabetic natural products such as medicinal plants that produce minimal or no side effects [4, 5]. Studies that reveal the mode of action of potential antidiabetic plants will definitely provide a scientific and systematic approach to the use of these plants as hypoglycemic agents [6].

Streptozotocin (STZ) is frequently used to induce experimental T1DM and its diabetic action results from its highly specific cytotoxic action on *β* cells by increasing production of oxygen free radicals which results in oxidative stress [7, 8].

*Balanites aegyptiaca* is a widely distributed African plant of medicinal interest. In Egyptian folk medicine, its fruit mesocarp is commonly used as an oral antidiabetic drug [9, 10].* Petroselinum sativum* is a cultivated variety of parsley and is traditionally used as carminative, abortifacient, and antihypertensive agent [11]. To the researchers' knowledge, there are no available studies that reveal the antidiabetic and antioxidant influences of* Balanites aegyptiaca* and* Petroselinum sativum* extracts on diabetes-induced experimental models or their possible hypoglycemic effects on normal animals. For these reasons, the current study has been undertaken to examine the possible antidiabetic and antioxidant effects of* Balanites aegyptiaca* fruits and* Petroselinum sativum *leaf aqueous extracts on STZ-induced diabetic (hyperglycemic) and normal (normoglycemic) rats with a view to transmitting the results for the human use.

## 2. Methods

### 2.1. Preparation of* Balanites aegyptiaca* Fruits and* Petroselinum sativum* Leaf Aqueous Extracts

*Balanites aegyptiaca* fruits and* Petroselinum sativum* leaves were purchased from local markets in Assiut Governorate. The pericarp of* Balanites aegyptiaca* fruits was cleaned and then prepared in the form of coarse powder.* Petroselinum sativum* leaves were washed with tap water, dried in the shade for one week, and stored in well-sealed cellophane bags. The dried leaves were powdered to be used for extract preparation. According to Gad and coworkers [6, 12], one kilogram of the dried powdered plant materials was extracted with four liters of boiling distilled water using percolation for 48 hours. The extracts were filtrated. Then, the filtrate was concentrated under reduced pressure by rotatory evaporation using a rotatory evaporator (Heidolph, Germany) at 40°C until the therapeutic residues were obtained. The therapeutic liquids were subjected to lyophilization using the freeze dryer (VirTis, USA) until fine powder materials were obtained and weighed to give 200 grams of dried powdered extract in the case of* Balanites aegyptiaca* and 150 grams in the case of* Petroselinum sativum*. Thus, the yielded extract was about 20% w/w and 15% w/w for* Balanites aegyptiaca* and* Petroselinum sativum*, respectively. The extracts were dissolved in distilled water before administration to diabetic and normal rats. The extracts were administered orally using an orogastric tube.

### 2.2. Experimental Animals

This study was conducted on adult male Wistar albino rats. Animals were obtained from the Animal House of the Faculty of Medicine, Assiut University. Their weight ranged between 150 and 200 grams at the beginning of the experiment. Rats were housed in groups in clean cages (five per cage) under standard laboratory conditions, including good aerated room with suitable temperature. Food and water were available* ad libitum*.

### 2.3. Induction of Experimental Diabetes

Diabetes was induced in overnight-fasted rats by single intraperitoneal injection of STZ (Sigma-Aldrich Company, USA) freshly dissolved in an ice-cold saline solution (0.9%) in a dose of 80 mg/kg BW [13]. Following STZ injection, rats were fed with glucose solution (5%) for 24 hours to avoid drug-induced hypoglycemia [8]. Blood glucose levels were determined three days after STZ injection using glucometer (Bionime, Taiwan) and the blood samples were taken from the tail vein. Rats with blood glucose range of 150–400 mg/dL were considered diabetic and included in this study [14].

### 2.4. Experimental Design

The sixty rats were randomly assigned to 6 groups (10 rats each). Three groups were assigned as normoglycemic groups in which the first one is normal control group (NC) and was given nothing except standard rat pellets and water; the second one was a* Balanites aegyptiaca* extract-treated normal (BAETN) group and was orally given* Balanites aegyptiaca* fruits aqueous extract (1.5 g/kg BW daily for 45 days) [6]; and the third one is* Petroselinum sativum* extract-treated normal (PSETN) group and was orally given* Petroselinum sativum* leaf aqueous extracts (2 g/kg BW daily for 45 days) [15].

The other three groups were assigned as hypoglycemic groups. The first one is diabetic control (DC) group in which rats received a single intraperitoneal injection of STZ. The second one is a* Balanites aegyptiaca* extract-treated diabetic (BAETD) group in which diabetic rats were treated orally with* Balanites aegyptiaca* fruits aqueous extract (1.5 g/kg BW daily for 45 days) [6]. The third group is* Petroselinum sativum* extract-treated diabetic (PSETD) group in which diabetic rats were treated orally with* Petroselinum sativum* leaf aqueous extracts (2 g/kg BW daily for 45 days) [15].

For all groups, 24 hours after the last dose of treatment, the live body weight (BW) was recorded using an electronic balance (A&D, Japan) and the rats were anesthetized and their blood specimens were individually collected from the retroorbital plexus of veins. The blood was centrifuged for 10 min at 3000 rpm and the sera were stored at −70°C until used. The necessary corrections of plasma parameters with plasma total protein levels (as an index of haemoconcentration) were made to eliminate the haemoconcentration effect [16].

### 2.5. Tissue Collection and Histomorphometric Study

The rats were anesthetized by ketamine (12 mg/kg BW) and then sacrificed by cervical dislocation. Liver and pancreas tissues were excised and rinsed in ice-cold physiological saline. The liver tissues were homogenized using a homogenizer (Ultra-Turrax T25 Basic, Werke, Germany) in 0.1 M Tris-HCl buffer at pH 7.4. The homogenate was centrifuged at 40000 revolutions/minute for 15 minutes in Heraeus Christ centrifuge to remove the nuclei and the cellular debris. The clear supernatant that contains cytosolic fraction was kept at −70°C to be used for assaying pyruvate kinase later on. The weight of the pancreas was recorded for each animal using a Mettler balance. Pancreatic weight per 100 g BW was calculated in the various experimental groups to make a functional comparison of weight loss and gain. The pancreases of each group were fixed in 10% neutral buffered formalin for 24 hours, and then they were processed for paraffin embedding. 5–7 *μ*m thick paraffin sections were stained with Harris' haematoxylin and eosin [17, 18]. The sections were examined and photographed on microscope with DP72 camera and cell software (Olympus, Japan). Images were labeled using Adobe Photoshop Version 8. Morphometrical studies were performed on the stained histological sections using an image analyzer (Leica Q500 MC, Leica, Germany). The sizes of 100 islets were measured in each group and the mean size was calculated.

### 2.6. Biochemical Analyses

Plasma glucose, total antioxidant capacity, and malondialdehyde and liver-pyruvate kinase levels were measured colorimetrically using spectrophotometer (Spectronic 21, Milton Roy Company, USA) [19], while the plasma insulin level was measured by ELISA reader (Stat Fax-200, Awareness Technology Company, USA) using specific ELISA kit.

### 2.7. Statistical Analysis

All data are expressed as mean ± SE deviation. Data were compared among groups using one-way analysis of variance (ANOVA) followed by Tukey posttest using Prism software (v4.00 for Windows, GraphPad Software, San Diego, CA). A *P* value of less than 0.05 was considered to represent a statistically significant difference.

## 3. Results and Discussion

### 3.1. Effects of* Balanites aegyptiaca* Fruits and* Petroselinum sativum* Leaf Aqueous Extracts on the Mean BW of the Normal and Diabetic Rats

**Figure 1:**
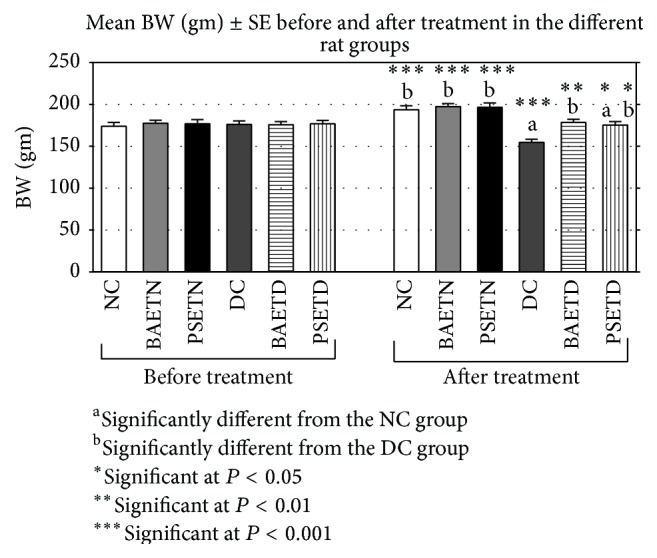
The effect of* Balanites aegyptiaca* fruits and* Petroselinum sativum* leaf aqueous extracts on mean BW (gm) in normal and STZ-induced diabetic rats (mean ± SE).

The mean final BW of the* Balanites aegyptiaca* extract-treated diabetic (BAETD) and* Petroselinum sativum* extract-treated diabetic (PSETD) groups were lower than that of the NC group, but the decrease was nonsignificant (Figure 1). Consistent with Jung and his colleagues [20], the diabetic control (DC) group is characterized by a significant reduction in its mean BW as compared with the NC group (Figure 1). Loss of BW could be explained by degradation of structural proteins, increased gluconeogenesis from muscle protein [21], and lipolysis of triglycerides [22] under the effect of experimental diabetes. It is noteworthy that, in the current study, along with reduction of BW in diabetic rats, polyphagia, polyuria, and polydipsia (data not shown) were also observed in parallel with a previous report by [23].

Compared with the DC group, the mean final BW of the BAETD and PSETD groups was significantly improved, but the BAETD group exhibited a better improvement in its mean final BW than that detected in the PSETD group (Figure 1). This effect on BW may be due to the ability of the extracts to reduce hyperglycemia which in turn corrects the aforementioned abnormalities including loss of BW [6]. However, in case of* Petroselinum sativum* leaf aqueous extracts, the results of the current experiment do not agree with the results from the study conducted by Yanardağ and his colleagues [24]. They showed a significant reduction in BW after treating the diabetic rats with* Petroselinum crispum* leaf aqueous extracts for 28 days due to the diuretic effect of the extract. This difference between our study and the study of Yanardağ and his colleagues might be due to the differences in the variety of parsley and/or the duration of the experiment.

### 3.2. *Balanites aegyptiaca* Fruits and* Petroselinum sativum* Leaf Aqueous Extracts Significantly (*P* < 0.001) Reduce the Elevated Plasma Glucose Level in the BAETD Group in Comparison with the DC Group

**Figure 2:**
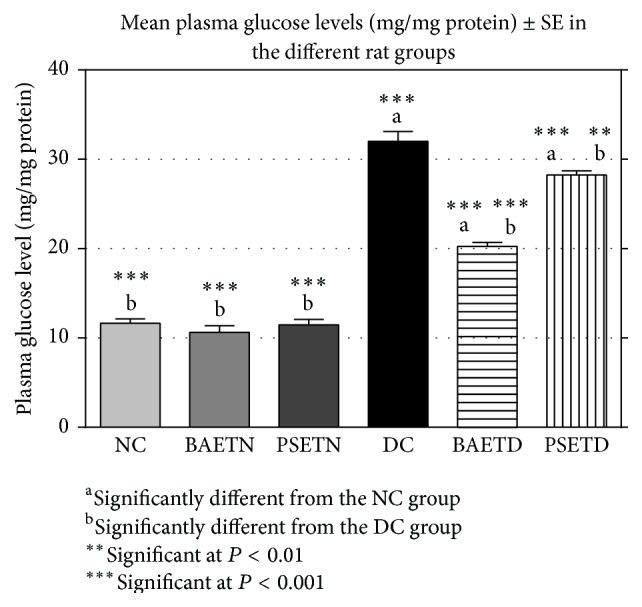
*Balanites aegyptiaca* fruits and* Petroselinum sativum* leaf aqueous extracts succeeded significantly (*P* < 0.001) in reducing the elevated mean plasma glucose level of the treated diabetic group in comparison with the DC group.

**Figure 3:**
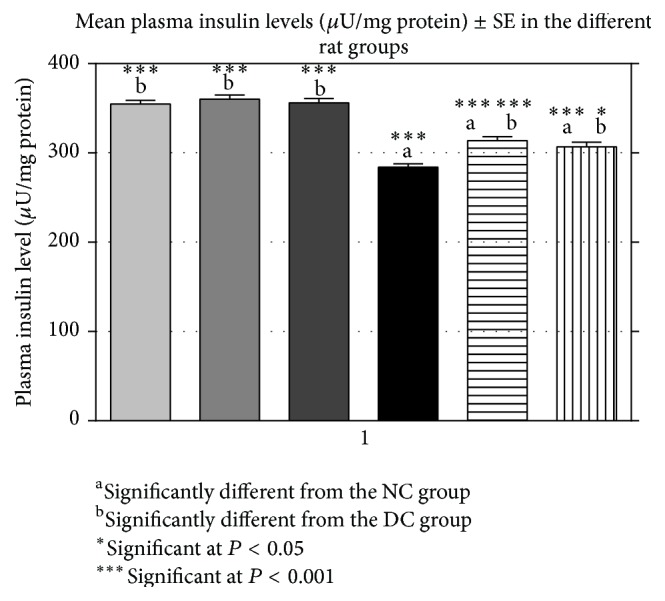
*Balanites aegyptiaca* fruits and* Petroselinum sativum* leaf aqueous extracts significantly (*P* < 0.001) increased the mean plasma insulin of the treated diabetic group in comparison with the DC group.

**Figure 4:**
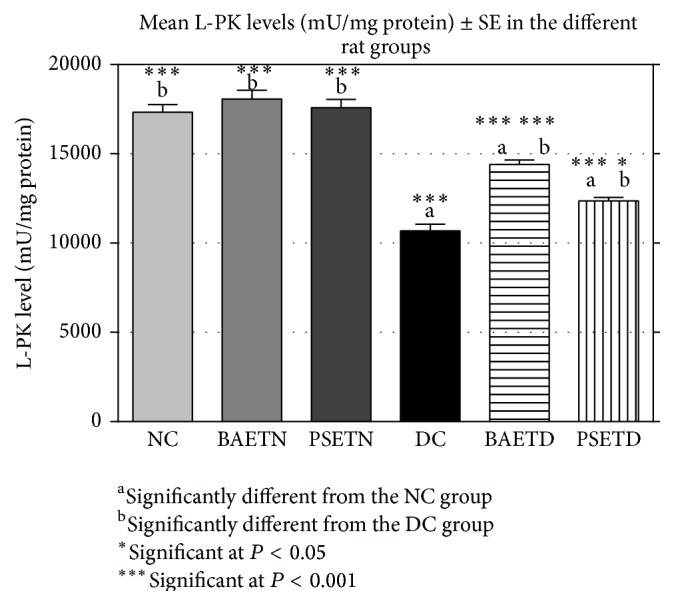
Effect of* Balanites aegyptiaca* fruits and* Petroselinum sativum* leaf aqueous extracts on the mean liver PK levels of normal and STZ-induced diabetic rats (mean ± SE).

A nonsignificant difference was found between the mean plasma glucose, insulin, and L-PK levels of the NC group and those of the BAETN and PSETN groups (Figures 2, 3, and 4). These results seem to be consistent with several reports about other plant extracts that failed to produce any changes in their levels in normal treated rats but caused a significant reduction in the blood glucose level and elevation in the blood insulin level in diabetic rats [25–27]. However, the finding of the present study is incompatible with the study of Shaw and his colleagues [5] who demonstrated a fast decline in the blood glucose level of normal rats after starch load with the* Balanites* extract suggesting that there is enhancement of glucose utilization by a single dose of* Balanites*. In agreement with Bolkent and colleagues [15, 23], nonsignificant changes were observed in the mean plasma glucose and insulin levels in normal rats that were given* Petroselinum sativum *leaf aqueous extracts. The absence of significant hypoglycemic activities for* Balanites aegyptiaca* fruits and* Petroselinum sativum* leaf aqueous extracts in normal rats in this study is a desirable feature as it has been noted that hypoglycemia causes a cascade of adverse effects [28].

STZ is the agent of choice for the induction of diabetic metabolic state in experimental animals [8]. In this work, STZ-induced diabetic rats were characterized by a significant increase in their mean plasma glucose level along with a significant decrease in their mean plasma insulin level in comparison with the NC rats as shown in several previous studies [20, 29].

The DC group was characterized by a highly significant (*P* < 0.001) increase in its mean plasma glucose level and a highly significant decrease in its mean plasma insulin and L-PK levels as compared with the NC group (Figures 2, 3, and 4).

*Balanites aegyptiaca* fruit aqueous extract succeeded in reducing significantly (*P* < 0.001) the elevated mean plasma glucose level of the BAETD group in comparison with the DC group. The* Balanites aegyptiaca* fruit aqueous extract showed more noticeable hypoglycemic effect than that of* Petroselinum sativum* leaf aqueous extracts (Figure 2). These results are in agreement with several previous studies [6, 10, 30, 31]. It was postulated that the hypoglycemic effect of the* Balanites aegyptiaca* extract is mediated through insulinomimetic activity [32], stimulation and potentiation of insulin secretion, increased insulin receptors affinity [33], enhancement of hepatic glycogen storage, suppression of hepatic gluconeogenesis, acceleration of glucose metabolism, and inhibition of intestinal glucosidase activity [6]. The hypoglycemic effect of* Petroselinum crispum* extract is believed to be related to the presence of flavonoids [24]. The antidiabetic potency of flavonoids has been highlighted in many reports and is attributed in part to their antioxidant and hypoglycemic effects.

### 3.3. *Balanites aegyptiaca* Fruits and* Petroselinum sativum* Leaf Aqueous Extracts Significantly (*P* < 0.001) Increased the Plasma Insulin Levels of the BAETD Group Compared with the DC Group

*Balanites aegyptiaca* fruits and* Petroselinum sativum* leaf aqueous extracts significantly (*P* < 0.001) increased the mean plasma insulin (Figure 3) of the treated diabetic group in comparison with the DC group. In agreement with El-Bayomy and colleagues [34, 35], administration of* Balanites aegyptiaca* fruit aqueous extracts to diabetic rats significantly increased their mean plasma insulin levels (Figure 3). These* in vivo* studies ran in parallel with an* in vitro* study conducted by Abdel-Moneim [33] which indicated that* Balanites aegyptiaca* stimulated the *β* cells of pancreatic islets to secrete insulin, potentiated the glucose stimulation of insulin secretion, and increases the number and affinity of insulin receptors and postreceptors in *β* cells. Yanardağ and his coworkers [24] suggested that* Petroselinum crispum *leaf extract did not increase insulin release from *β* cells of the pancreas and that inhibition of gluconeogenesis and direct stimulation of glycolysis may be involved in the mechanism of its hypoglycemic action. The apparent differences in the results of the present study and the previous one could be explained on the basis of the dissimilarity in the examined varieties of parsley that might affect the presence and/or concentrations of the active constituents. In addition, the methods of extraction are also different: evaporation under reduced pressure followed by lyophilization in this study versus evaporation under reduced pressure only in the preceding one.

### 3.4. *Balanites aegyptiaca* Fruits and* Petroselinum sativum* Leaf Aqueous Extracts Significantly (*P* < 0.001) Increase the Mean L-PK Levels of the BAETD Group in Comparison with the DC Group

L-PK catalyzes the conversion of phosphoenolpyruvate to pyruvate and this represents the last irreversible steps of glycolysis in the liver [36]. It was suggested that the increase in the mean level of the L-PK is directly responsible for increased utilization of glucose in the liver [37] and it occurred most probably as a result of the insulinotropic action of the extract [38] by stimulating the transcription of the gene encoding L-PK. The rise in the insulin level plays an indirect role in L-PK activation by antagonizing the action of glucagon to stimulate protein kinase A-mediated phosphorylation of the enzyme [39].

In the present study, we observed a significant decrease in the mean L-PK level of diabetic rats compared with normal untreated rats (Figure 4). This might be due to the reduction in the transcription of gene encoding L-PK in the insulinopenic diabetic state [39].

BAETD group showed a significant increase in its mean L-PK level in comparison with the DC group (Figure 4). This increase was greater than that detected in the PSETD group. Similar to the present study, other herbal extracts had been shown to improve the diabetic condition by increasing L-PK levels in diabetic rats [30, 38]. In contrast, Kamel [10] found that oral administration of both saponin mixture and polysaccharides (isolated from* Balanites aegyptiaca* mesocarp aqueous extract) after six hours did not exert any effect on liver hexokinase (another key regulatory enzyme of glycolysis) activity in diabetic mice.

With respect to the influence of* Petroselinum sativum* leaf aqueous extracts on the mean level of the L-PK, no data are available about their effect on this enzyme or even any one of the glycolytic enzymes. Nevertheless, the finding of this study supports the hypothesis that direct stimulation of glycolysis by* Petroselinum crispum* extract may be involved in the mechanism of its hypoglycemic action [24].

It was suggested that the increase in the mean level of the L-PK following extracts' dosing occurs most probably as a result of the insulinotropic action of the extract [38] by stimulating the transcription of the gene encoding L-PK. The rise in the insulin level plays an indirect role in L-PK activation by antagonizing the action of glucagon to stimulate protein kinase A-mediated phosphorylation of the enzyme [39].

### 3.5. *Balanites aegyptiaca* Fruits and* Petroselinum sativum* Leaf Aqueous Extracts Significantly (*P* < 0.001) Increased Plasma TAC Levels and Significantly (*P* < 0.01) Decreased Plasma MDA Levels of the BAETD Group Compared with the DC Group

**Figure 5:**
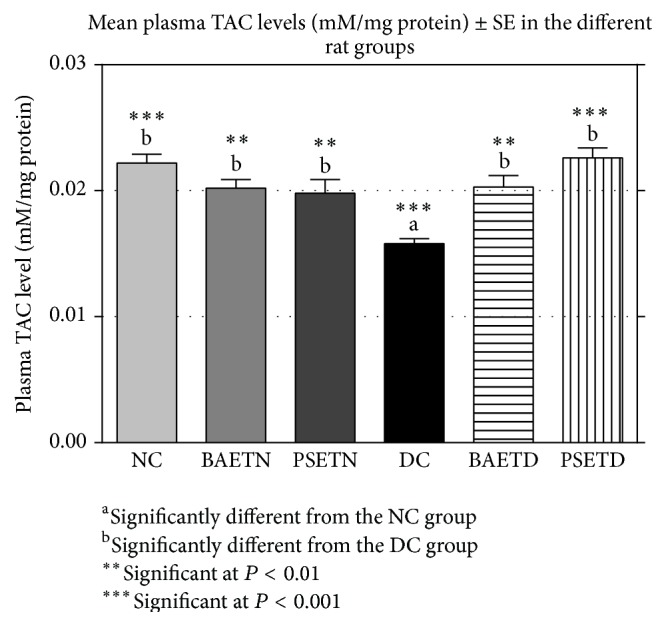
Oral administration of either* Balanites aegyptiaca* fruit aqueous extract or* Petroselinum sativum* leaf aqueous extracts to the diabetic rats produced a significant (*P* < 0.01) increase in their mean plasma TAC levels when compared with the diabetic control rats.

In the present study, the mean plasma TAC level in the DC group was significantly decreased in comparison with the NC group (Figure 5). This finding coincides with the other studies which reported that diabetes was associated with reduced levels of antioxidant activity in the blood and tissues of diabetic animals [40, 41]. The reduced mean plasma TAC level in the DC group might indicate that there was an imbalance between free radical formation and antioxidant protection as a consequence of increased utilization of endogenous antioxidants in response to the elevated levels of free radicals [16].

STZ treatment significantly increased lipid peroxides and decreased antioxidant enzyme activities in the plasma of rats, confirming that STZ-induced diabetes is accompanied by increased generation of ROS [42], which in turn induced lipid peroxidation [43]. The results of the present experiment agreed with the previous data as well as with the hypothesis of Meenu and his colleagues [44] who postulated that hyperglycemia resulted in the generation of free radicals, which can exhaust antioxidant defence, thereby leading to the disruption of cellular function and oxidative damage to the membranes, and enhance the susceptibility to lipid peroxidation.

**Figure 6:**
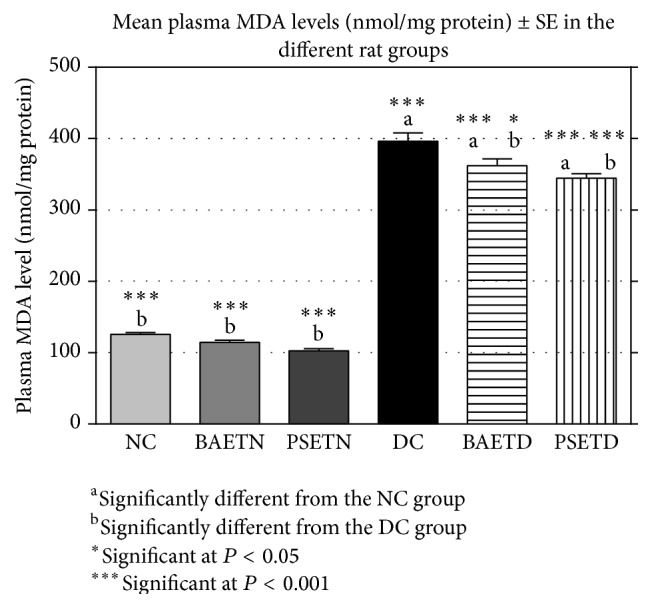
Oral administration of either* Balanites aegyptiaca* fruit aqueous extract or* Petroselinum sativum* leaf aqueous extract to the diabetic rats produced a significant (*P* < 0.01) decrease in their mean plasma MDA levels when compared with the diabetic control rats.

Oral administration of either* Balanites aegyptiaca* fruit aqueous extract or* Petroselinum sativum* leaf aqueous extract to the diabetic rats produced a significant (*P* < 0.01) increase in their mean plasma TAC levels (Figure 5) and a significant (*P* < 0.01) decrease in their mean plasma MDA levels (Figure 6) when compared with the diabetic control rats. Supplementation of* Petroselinum sativum* leaves aqueous extract to the diabetic rats resulted in a greater enhancement in their mean plasma TAC and MDA levels than* Balanites aegyptiaca* fruit aqueous extract.

In the present study, the mean plasma MDA level was decreased significantly in the PSETD group compared with the DC group. This is consistent with a significant reduction in the elevated plasma level of MDA in the cisplatin and CCl_4_ poisoned rats following administration of* Petroselinum sativum* leaf extracts [12, 45]. The* in vivo* studies were confirmed by another* in vitro* study carried out by Al-Mamary [46] who found that* Petroselinum sativum* leaves juice inhibited liver homogenate oxidation mediated by the ferric sulphate/ascorbate system.

The antioxidant activities of* Balanites aegyptiaca* fruits and* Petroselinum sativum* leaf aqueous extracts in diabetic rats that were observed in this study may be secondary to their hypoglycemic effects as well as the presence of some phytochemical antioxidant agents. It was postulated that these activities may be due to enhancement of antioxidant enzyme synthesis by acting on the antioxidant response elements in the enhancer region at the promoter site of the gene that codes for the enzymes [47].

### 3.6. Histomorphological Study

In the BAETN and PSETN groups, the mean weight of pancreatic tissue and the size of the islets of Langerhans were nonsignificantly different from those of the NC group. As far as the researchers are concerned, there were no published studies in the literature focusing on the influence of any of these extracts on the pancreas weight in normal laboratory animals. Nevertheless, evidence from the histopathological part of this study indicated that both extracts were unable to significantly increase the size of the pancreatic islets in the treated normal rats which indicates that the growth stimulation effect of both herbal extracts can only appear in diabetic rats, not in normal ones.

**Figure 7:**
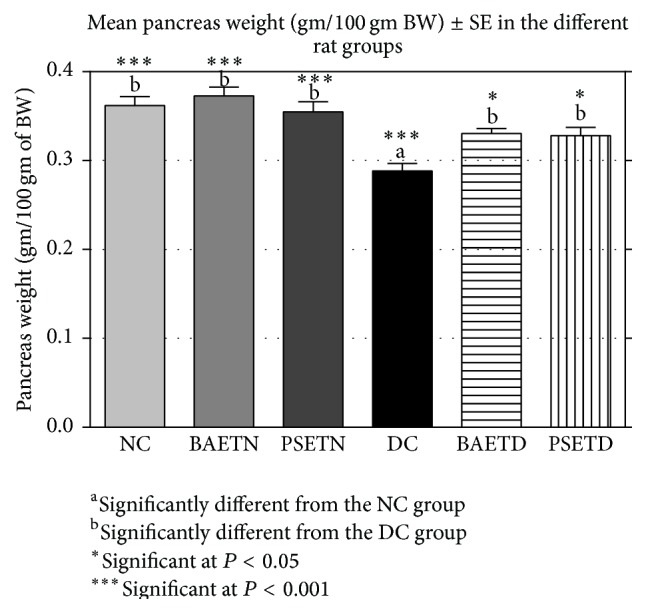
Effects of* Balanites aegyptiaca* fruits and* Petroselinum sativum* leaf aqueous extracts on the mean pancreas weight (gm/100 gm of BW) in normal and STZ-induced diabetic rats (mean ± SE).

As shown previously by Bangar and colleagues [48, 49], we found a significant decrease in the mean weight of the pancreas of the diabetic rats compared with the mean weight of the pancreas of untreated normal rats (Figure 7).

Some researchers used the size of pancreatic islets as an index for the evaluation of the antidiabetic effects of different extracts [48, 50]. In addition, we found that the reduction in the mean weight of the pancreas was accompanied by a significant decline in the size of pancreatic islets in STZ-induced diabetic rats and by an obvious degeneration and necrosis of *β* cells (Figure 9(d)). A previous study conducted by Heidari and his colleagues [51] attributed the reduction in the weight of the pancreas to the disruption and the disappearance of pancreatic islets and the selective destruction of *β* cells by the diabetogenic action of STZ.

**Figure 8:**
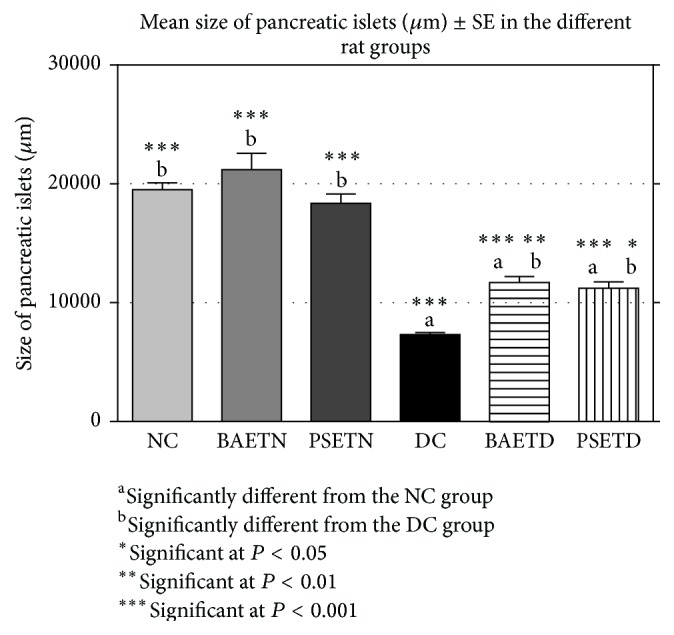
Administration of* Balanites aegyptiaca* fruits and* Petroselinum sativum* leaf aqueous extracts to the diabetic rats exhibited a significant increase in the mean size of the islets of Langerhans as compared with the DC group.

Administration of* Balanites aegyptiaca* fruits and* Petroselinum sativum* leaf aqueous extracts significantly increased the mean weight of the pancreas in STZ-induced diabetic rats (Figure 7) and the mean size of the islets of Langerhans (Figure 8) as compared with the DC group. This has been associated with improvement in the histopathological pictures of their pancreas. To our knowledge, all the previous studies have not focused on the changes in the pancreas weight following administration of either* Balanites aegyptiaca* extracts or* Petroselinum sativum* extracts on the diabetic animal models. However, the data of the current work are in agreement with many researchers who reported that several other plant extracts succeeded in restoring the pancreas weight of diabetic rats by repairing or regenerating pancreatic *β* cells [48, 52].

**Figure 9:**
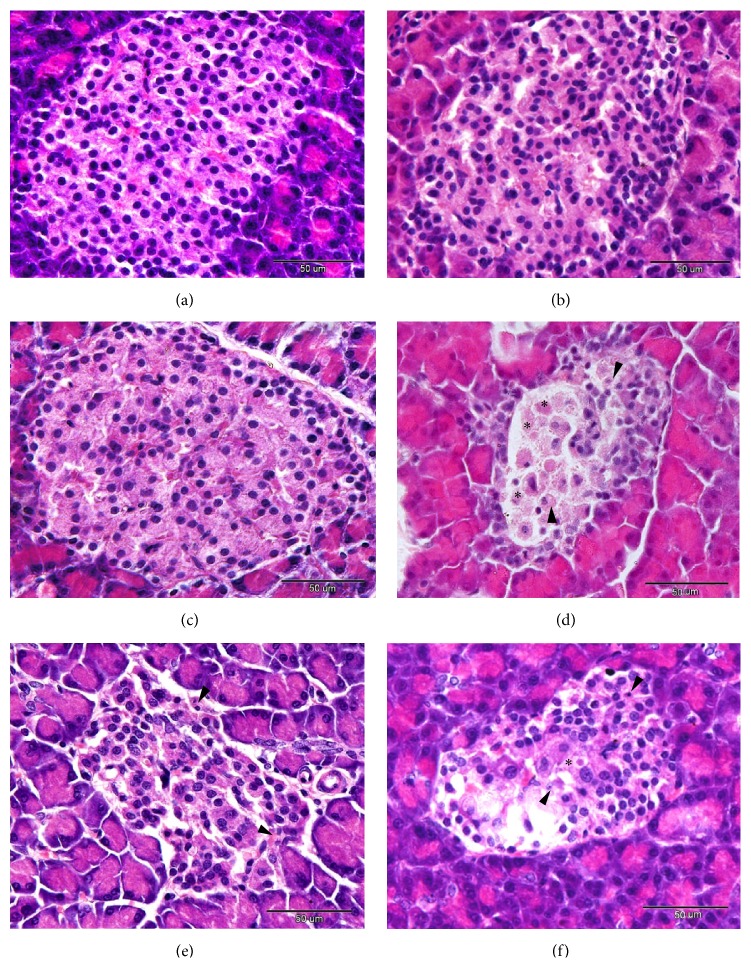
((a)–(d)) A photomicrograph of the pancreas of NC group (a), BAETN group (b), and the PSETN group (c). The diabetic pancreas (d) showed severe necrotic and degenerative changes (arrowheads). Residues of the cytoplasm and the nuclei were also seen (asterisks). In the BAETD and PSETD groups ((e) and (f), resp.), the degenerative and necrotic changes were markedly lower than that observed in the DC group, H&E staining. Scale bar = 50 *μ*m.

The histopathological pictures of the pancreas in the BAETN and PSETN groups were similar to that of the NC group. Severe degenerative and necrotic changes were observed in the islets cells of the DC group when compared with the control group (Figures 9(a) and 9(d)). In addition, the nuclei of the pancreatic cells showed pyknosis and karyolysis. Disappearance of some cells nuclei and residues of the nuclei and destroyed cells was also seen. Following the administration of* Balanites aegyptiaca* fruits and* Petroselinum sativum* leaf aqueous extract to the diabetic rats, most of the cells appeared normal (Figures 9(b) and 9(e)), while in the PSETD group, few degenerative and necrotic changes were still found in the pancreatic islets cells (Figures 9(c) and 9(f)). The pancreatic tissue in the BAETD group showed a better improvement than that observed with the PSETD group.

The exact mechanism by which the extracts regenerated islet *β* cells and increased the size of pancreatic islets at the cellular level has not been examined in this study, but some hypothesis can be put forward. It is well known that *β* cell apoptosis is a common feature of T1DM [53]. In fact, both* Balanites aegyptiaca* [54] and* Petroselinum sativum* [11], like many herbal extracts, are rich in flavonoids. The flavonoid genistein reduced *β* cell apoptosis in pancreatic islets, increased the number of insulin-positive *β* cells in the islets, promoted islet *β* cell survival, and preserved islet mass [55]. It is clear by now that alternative approaches for treatment of T1DM include the stimulation of regeneration of endogenous pancreatic *β* cells. Three different challenges to meet these alternative approaches include protection of the new *β* cells from autoimmunity and allorejection [56], the endogenous replenishment of pancreatic *β* cells [57], and the transdifferentiation from pancreatic acinar cells [58]. It was revealed that flavonoid rich fractions of* Oreocnide integrifolia* leaves have an enhancing influence on islet neogenesis and greater *β* cell regeneration in pancreatectomized BALB/c mice [59].

## 4. Conclusion

This study showed that* Balanites aegyptiaca* and* Petroselinum sativum* aqueous extracts have antidiabetic and antioxidant effects on the diabetic rats. These extracts can be potentially used with insulin therapy to minimize its side effects and to improve the treatment of T1DM and probably other oxidative stress-associated diseases.
